# Estimating disease vector population size from citizen science data

**DOI:** 10.1098/rsif.2021.0610

**Published:** 2021-11-24

**Authors:** Tam Tran, W. Tanner Porter, Daniel J. Salkeld, Melissa A. Prusinski, Shane T. Jensen, Dustin Brisson

**Affiliations:** ^1^ Department of Biology, University of Pennsylvania, Philadelphia, PA 19104, USA; ^2^ Pathogen Genomics Division, Translational Genomics Research Institute, Flagstaff, AZ 86005, USA; ^3^ Department of Biology, Colorado State University, Fort Collins, CO 80523, USA; ^4^ Bureau of Communicable Disease Control, New York State Department of Health, Albany, NY 12237, USA; ^5^ Department of Statistics, The Wharton School of the University of Pennsylvania, Philadelphia, PA 19104, USA

**Keywords:** citizen science, community science, Lyme disease vector, population trends

## Abstract

Citizen science projects have the potential to address hypotheses requiring extremely large datasets that cannot be collected with the financial and labour constraints of most scientific projects. Data collection by the general public could expand the scope of scientific enquiry if these data accurately capture the system under study. However, data collection inconsistencies by the untrained public may result in biased datasets that do not accurately represent the natural world. In this paper, we harness the availability of scientific and public datasets of the Lyme disease tick vector to identify and account for biases in citizen science tick collections. Estimates of tick abundance from the citizen science dataset correspond moderately with estimates from direct surveillance but exhibit consistent biases. These biases can be mitigated by including factors that may impact collector participation or effort in statistical models, which, in turn, result in more accurate estimates of tick population sizes. Accounting for collection biases within large-scale, public participation datasets could update species abundance maps and facilitate using the wealth of citizen science data to answer scientific questions at scales that are not feasible with traditional datasets.

## 1. Introduction

The rise of public participation in data collection [1] provides unprecedented opportunities for scientific research. Voluntary public participation in scientific research—often referred to as citizen science—allows rapid, inexpensive and massive-scale data collection across expansive temporal and geographical ranges [2]. Public involvement in data collection alleviates researchers from the financial and labour constraints that often narrow the power, scale and generalizability of individual scientific projects. For example, citizen science data have monitored weather patterns and bird populations across North America for over a century [3,4], discovered new planets [5], classified galaxies [6] and crowd-sourced biodiversity observations [7,8]. These and many similar projects were made possible by the immense volumes of data collected by millions of participants [1,9]. However, the variation in participation and effort among untrained collectors has led some to question the reliability and accuracy of citizen science datasets [9]. The inability to identify and resolve citizen science data collection inconsistencies may result in inaccurate representations of the system being studied [2,10,11].

The dependability of citizen science data is often inversely related to the number of data collectors and the magnitude of the data collected. Citizen science projects can range from the involvement of a few carefully directed individuals to many thousands of independent contributions from the general public, the latter being the focus of this paper [12]. Training and guidance of participants by researchers improve citizen science data accuracy but often at the cost of a reduction in the number of participants and the scope of the study [9]. For instance, smaller scale projects can standardize datasets by accounting for the variance in data collection efforts or success among participants that result from different individual skill levels or day-of-collection factors that cause inconsistencies in data quality [9]. By contrast, variation among volunteers from the general public cannot be recorded and results in difficulties discerning discrepancies in data collection [10,13]. Yet, identifying and accounting for dataset inconsistencies from the untrained general public could expand the possibilities of scientific enquiry by harnessing past and future large-scale citizen science datasets.

Dependable citizen science datasets can address scientific questions that are beyond what is currently feasible. For example, the eBird citizen science dataset, which includes observations by 670 000 people across the world [14], has been used to describe the distribution and relative abundance of over 800 bird species [15,16]. However, large-scale citizen science datasets are rarely evaluated for data quality ([17], but see [16]), in part because of the lack of comparable datasets, despite the wealth and value of citizen science data [12]. Assessing the accuracy of large-scale citizen science datasets can be accomplished by pairing citizen science datasets with datasets built using rigorous data collection protocols that have similar temporal and spatial scopes. Ecological citizen science datasets are ideal for validating the value of public participation over large spatial and temporal ranges because the long-standing societal interest in the natural world has contributed to extensive species distribution collections [2,18,19]. Citizen science projects that capture information on population dynamics as reliably as data collected by trained scientists would consequently reduce the challenge of large-scale ecological data collection [2,10]. Validated citizen science datasets have the potential to depict population dynamics more accurately over a larger geographical expanse, such as at a state or nationwide scale, which would improve the generalizability of findings [1,20,21]. Identification of a framework to validate and quantitatively account for the biases within citizen science data collections could improve the reliability of citizen science across disciplines.

Here, we investigate whether a large citizen science dataset corresponds with scientifically rigorous data collected over a large geographical area and across years. We focus on collections of the black-legged tick, *Ixodes scapularis*, which has garnered significant public interest as the primary vector of several human diseases, including babesiosis, anaplasmosis, Powassan encephalitis and Lyme disease [22]. The public health burden of the pathogens transmitted by this tick has resulted in widespread surveys by the scientific community, public interest in participating in tick surveillance and tick identification and pathogen testing services available to the public. The scale of the *I. scapularis* tick collections by both the scientific community and citizen science projects has provided an ideal scenario to assess the accuracy of large-scale citizen science data. We demonstrate that citizen science can be used to characterize vector population abundance over one of the largest spatial extents yet. Identifying and resolving inconsistencies in the spatial and temporal variability of annual population sizes found in public tick collection datasets would improve the accuracy and applicability of citizen science data. These data could be used to update species abundance maps and serve as an important ecological tool to address a significant public health issue.

## 2. Material and methods

### 2.1. Study system

New York State (NYS) has one of the highest numbers of Lyme disease cases in the country (constituting over 10% of cases in the USA), and *I. scapularis* has undergone major population expansion in recent decades within the state [23–25]. Ticks are present in all counties, although tick abundance and the timing of tick population establishment are variable [26]. NYS is the fourth most populous state in the country, with a population of nearly 20 million people over 62 counties across a landmass that would be ranked as a medium-sized country [27]. Counties are heterogeneous in size and population density, ranging from 3 to 70 000 people per square mile [28]. The state is ecologically diverse and contains a wide variety of habitats from wetlands and mountainous regions to large cities and farmlands [29].

### 2.2. Tick data from citizen science

A nationwide free tick identification and pathogen testing service was made available to the public through Northern Arizona University. The programme was advertised to the public through a website and an initial public relations campaign. The programme was conducted without interruption from January 2016 to December 2017 [30]. Only *I. scapularis* submissions were included in these analyses. The submission date corresponded with the tick phenology observed in the field, with submission peaks corresponding to nymphal and adult activity in the spring and autumn, respectively. There was a mean of 1.27 ticks sent per submission, with submission sizes ranging from 1 to 17 ticks. Citizen science data were summarized as the total ticks submitted by each NYS county for each year. Ticks classified as *I. scapularis* from NYS were submitted from March 2016 to December 2017, with 447 ticks in 2016 and 697 ticks in 2017 (electronic supplementary material).

### 2.3. Tick data from active surveillance

**Figure 1.**
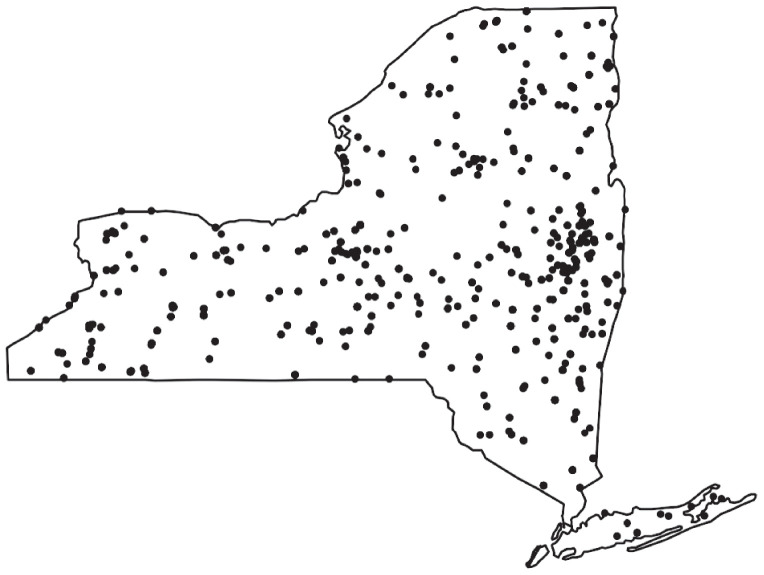
Active tick surveillance in 2016–2017: each point represents a collection site where a minimum of 1000 m was surveyed at each site.

The New York State Department of Health (NYSDOH) directs an ongoing tick surveillance programme throughout NYS that began in 2003 [31–33]. New York City (NYC) has an autonomous Department of Health and Mental Hygiene that independently directs its tick collections on publicly accessible land. The five counties that constitute NYC were thus excluded from these analyses, as there were no available active tick surveillance data. Locations for tick surveillance are predetermined before collection and deliberately consist of regions of suspected tick presence and absences, including locations beyond the current known geographical distribution of *I. scapularis* in NYS. Site selection is not biased towards regions with higher tick abundance nor towards convenience sampling. Sites are sampled non-uniformly from April to early December, with some sites being visited multiple times annually. Collections followed a uniform tick sampling protocol at all sites, consisting of standardized dragging, flagging and walking surveys using 1 m^2^ of white flannel or canvas. Over 27 000 *I. scapularis* nymphs have been collected across 612 sites from 2003 to 2017 (figure 1). There were 3074 and 4276 nymphal ticks collected in 2016 and 2017, respectively, over 377 sites (electronic supplementary material).

### 2.4. Estimated tick population sizes for New York State

The more than 15-year collection by the NYSDOH is one of the most extensive geospatial active surveillance efforts for ticks and provides the best estimate of actual tick density across NYS as collections accounted for distance surveyed and collection effort. This dataset was the basis for a validated, two-part model described in Tran *et al*. [23] that accurately estimated tick population sizes as a composite of both the probability of occurrence and the population size [23]. The prediction accuracy to a dataset collected in a future year which included previously unvisited locations was 80% for the presence model and 75% for the abundance model. Using the composite of the occurrence and presence models, tick population sizes throughout NYS were calculated as a raster map for 2016 and 2017. The results were then summarized as the estimated total number of ticks by county for each year, the same units as the citizen science tick submission data, for statistical analysis as described below (electronic supplementary materials).

### 2.5. Collector-associated factors

A literature review identified factors with a potential to influence participation in a tick submission citizen science programme. The citizen science programme did not solicit any information from participants, aside from county of tick exposure, such that individual-level human characteristics could not be used as factors to account for the variation in collector participation or effort. Thus, all identified factors that could potentially account for variation among participants were compiled at the county level to correspond in scale to the citizen science dataset. The identified factors can be grouped into three broad categories, including human demography, level of experience with Lyme disease and human activity level. Human demographic factors, each of which has been associated with human Lyme disease risk, include median household income, population size, poverty level, race, education and age distribution [34]. Summary statistics of relevant demographic factors were compiled from the United States Census [35]. Proxies for the level of experience with Lyme disease include local Lyme disease incidence and Google search trends [36–38]. Lyme disease incidence data are summarized as the number of cases per 100 000 people in each county [39]. Google Trends data ranks the proportion of annual Google searches for the term ‘Lyme disease’ in each region, which required grouping the 62 counties into 10 regions during analyses [40]. The mean annual temperature was identified as a potential predictor of human outdoor activity which could correlate with exposure to ticks [38,41]. It is important to note that temperature also impacts tick densities and activity and is unlikely to explain much of the variability in human outdoor activity behaviour [42]. The mean annual temperature for each county was obtained from the US Climate Divisional Database through the National Oceanic and Atmospheric Administration (NOAA) [43]. The correlation between these different factors varies, ranging between −0.74 and 0.78 using Pearson's *r* coefficient.

### 2.6. Analysis

Spearman rank correlation was used to explore the association between the total number of ticks per county in 2016 and 2017 estimated by citizen science data and active surveillance data. In addition, linear regression models were used to assess the impact of each collector-associated factor on biases in the citizen science dataset. Briefly, the response variable in all the regression models was the natural log-transformed annual tick abundance from each county as estimated from active surveillance while the predictors included log-transformed tick abundance estimates from citizen science and each collector-associated factor. Linear regression models were fitted using the iteratively reweighted least-squares method. Estimates from the regression models (also on the natural log scale) were then compared with total tick estimates from NYSDOH to determine how well models using citizen science data predict tick abundance in NYS. Comparison of the predictive accuracy of regression models with the addition of each predictor was based on root-mean-square-error (RMSE), R-squared (*R*^2^) and Akaike information criteria (AIC) (electronic supplementary materials). The full model and the most parsimonious model—which excludes poverty, Google trends, % white population and medium age—have similarly accurate predictive power (*R*^2^ = 0.63; RMSE = 0.45 versus *R*^2^ = 0.61; RMSE = 0.46). However, all analyses presented focus on the full model where possible to avoid suggesting causal relationships between any particular predictor and the number of ticks collected.

## 3. Results

**Figure 2.**
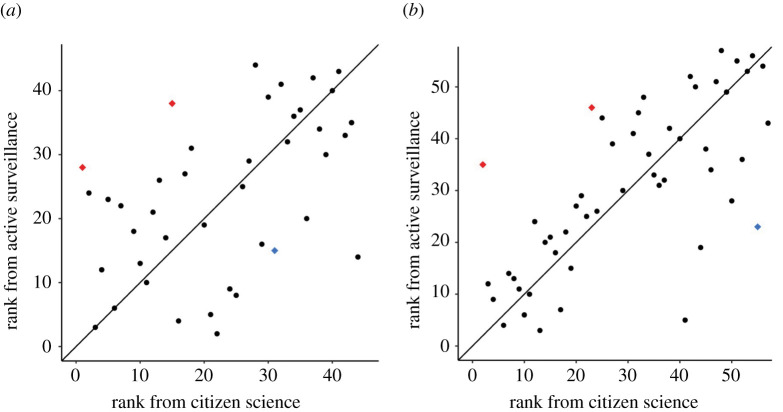
The tick population size in each county correlates with the number of ticks collected by the public in both 2016 (*a*) and 2017 (*b*). The number of ticks submitted by the public ranked across counties (*x*-axis) was similar to the rank of tick population sizes estimated from active surveillance (*y*-axis) with the diagonal line showing perfect correspondence. The consistency in the discrepancies between the datasets across years can be illustrated using data from Cayuga and Nassau counties (red points) and Warren county (blue points) as examples. That is, counties such as Cayuga and Nassau have large tick populations but few ticks were submitted from the public. By contrast, counties such as Warren have smaller tick populations but high tick submissions from citizen science. The datasets corresponded more strongly in 2017 (Spearman *ρ* = 0.71, *p* = 4.1 × 10^−9^) than in 2016 (*ρ* = 0.53, *p* = 2.7 × 10^−4^). Ticks were submitted by the public from fewer counties in 2016 (43 counties) than in 2017 (56 counties), resulting in different axis lengths.

The number of ticks collected by the public in each county in NYS is strongly correlated with the size of the tick population as determined by active surveillance (figure 2). That is, the largest numbers of ticks were submitted by citizens of the counties with the largest tick populations, and few ticks were submitted by citizens of counties with smaller tick populations. The congruity between the citizen science data and the data from active surveillance was consistent between years, although correlations were stronger in 2017 owing to tick submissions from more counties (43 versus 56). There were, however, several counties with relatively small tick populations from which large numbers of ticks were submitted by the public and several counties with larger tick populations from which few ticks were submitted. As examples, Cayuga and Nassau counties have relatively large tick populations but few ticks were submitted by citizens from these counties (figure 2, red points); by contrast, Warren county hosts a small tick population, but many ticks were submitted by citizens (figure 2, blue points). The counties in which the citizen science data did not agree with the active surveillance data in 2016 showed the same discrepancy in 2017 in both direction and magnitude.

**Figure 3.**
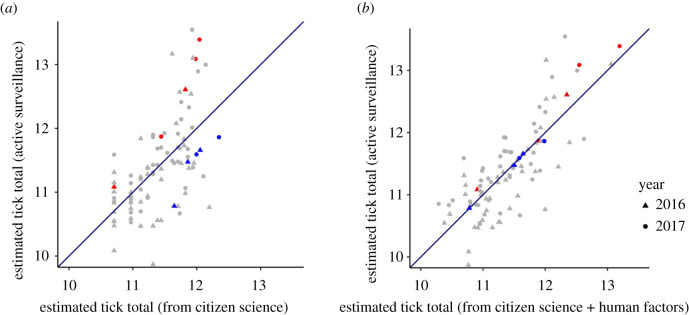
Collector-associated factors can rectify consistent errors in citizen science datasets. Models built with tick submissions from citizen science as predictors (*x*-axis) can predict actual tick population sizes, as estimated by active surveillance (*y*-axis). A model using only citizen science data (*a*) exhibits moderate accuracy, with evenly distributed errors as the tick population sizes are both underpredicted and overpredicted. The addition of nine collector-associated factors without Lyme disease corrects biases in citizen science data resulting in a model (*b*) that accurately predicted tick abundance. Collector-associated factors improved underpredictions and overpredictions. For example, a randomly selected set of sites that are overpredicted by the citizen science data (the red points in (*a*)) are much more accurately predicted by the full model (red points representing the same counties are much closer to the diagonal line in (*b*)). Similarly, a randomly selected set of sites that are underpredicted by the citizen science data (blue points in (*a*)) are much more accurately predicted by the full model (blue points in (*b*)). Both axes represent total ticks per county on the natural log scale (e ≍ 2.718) for 2016 and 2017 estimates.

The number of ticks submitted by the public from each county accounts for 37% of the variance in tick population size across counties (figure 3*a*; table 1, citizen science model). That is, the number of ticks submitted by the public from each county can estimate the underlying tick population sizes of each county with modest accuracy. However, the regression model including only citizen science data as a predictor consistently underestimates tick population sizes in areas with many ticks. Additionally, this model has proportionally large errors that are evenly distributed in counties with fewer ticks, owing to the coarseness of the citizen science estimates from counties that submitted fewer than five ticks.

**Table 1.** Regression models predicting the estimated number of ticks per NYS county.

description of model	RMSE	*R*^2^	AIC
citizen science model: *ticks collected from citizen science only*	0.58	0.37	172
**full model without Lyme disease:** *ticks collected from citizen science* *+* *all nine collector-associated factors but Lyme disease*	**0.45**	**0.63**	**139**
*all models below include citizen science tick submissions as a predictor*			
median household income	0.51	0.52	148
mean temperature	0.51	0.51	149
population	0.53	0.48	155
% below poverty	0.55	0.44	163
Google trends	0.55	0.43	164
% white population	0.56	0.43	164
% bachelor's degree or higher	0.57	0.39	170
% children (0–14 years old)	0.57	0.39	170
county's median age	0.58	0.38	172
*Lyme disease models include citizen science tick submissions as a predictor*			
Lyme disease incidence rate	0.58	0.38	172
full model with Lyme disease: *ticks collected from citizen science* *+* *all nine collector-associated factors with Lyme disease*	0.44	0.64	138

**Figure 4.**
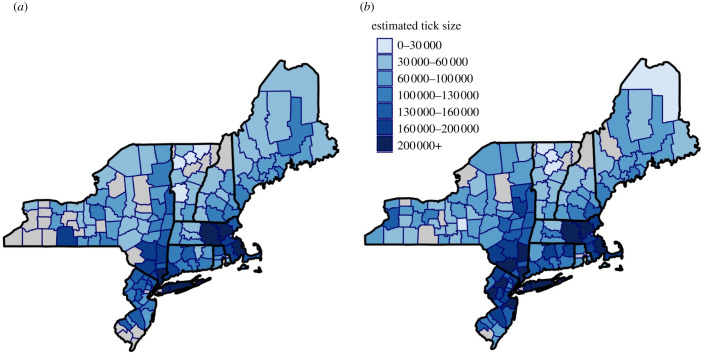
Models built on citizen science tick submissions and collector-associated factors can be extrapolated across the northeastern USA ((*a*) 2016 and (*b*) 2017). The high predictive accuracy of the models in NYS suggests a powerful tool to estimate *I. scapularis* population sizes in the counties of nearby states. Predictions from the full model without Lyme disease capture tick population size variability both among counties and between years in the same counties across northeastern states. Tick population sizes are represented as a heat map, with darker colours representing larger population sizes. Grey represents counties with no citizen science tick submissions. Predictions were made using the full model without Lyme disease with the exception of the Google Trends predictor owing to the lack of these data at the appropriate resolution.

A linear model that includes citizen science data and nine collector-associated factors as predictors accounts for 63% of the variation in tick population sizes among counties (table 1, full model without Lyme disease). Several of the predictors improved the fit of the model only marginally when included as the only collector-associated factor with tick submissions from citizen science (i.e. median age and the proportion of children in a county), while other predictors had much larger effects on model fit (i.e. income and temperature). Including county population size and household income in the linear model substantially improves underestimates of tick population sizes while including county poverty levels improves overestimates (red and blue points in figure 3, respectively). A 10-fold cross-validation evaluation of the full model without Lyme disease resulted in a similar model fit (*R*^2^ = 0.61; RMSE = 0.49), suggesting that these results are robust to overfitting. Model residuals of this full model without Lyme disease showed no departure from normality and no evidence of autocorrelation (figure 3*b*). The accuracy of this statistical model enabled predictions to counties in nearby states (figure 4).

## 4. Discussion

The immense quantity of citizen science data can capture population sizes in ecological systems across large spatial scales—information which is essential to conservation, agriculture and public health efforts [21]. However, citizen science data may be distorted by systematic inconsistencies in data collection [9,10]. The public tick collection data coarsely correspond with scientifically collected data at the county level across NYS. Further, the discrepancies and congruencies were consistent between years, suggesting consistency in participation biases (figure 2). These consistent participation biases can be accounted for in statistical models using collector-associated epidemiological and human behavioural information. Statistical models that include citizen science data and any single collector-associated predictors examined improve the accuracy of tick population size estimates over the citizen science data alone, although several resulted in only marginal improvements. Statistical models including all of the collector-associated factors investigated in this study resulted in highly accurate estimates of tick population sizes. Including collector-associated information to model large-scale animal population sizes leads to broad, new possibilities of harnessing the wealth of citizen science data to address important ecological questions and monitor populations in real time.

Systematic biases in citizen science datasets must be identified and resolved in order to rigorously assess scientific hypotheses. For example, citizen science tick collections generally measure tick exposure—where and when humans come into contact with ticks—whereas active surveillance assesses actual tick density by collecting in diverse settings, including locations uncommonly visited by the public. Citizen science datasets can complement or expand active surveillance data or, more importantly, address scientific hypotheses after demonstrating that the data accurately represent the underlying tick density and are scaled appropriately [30]. However, identifying systematic errors in large citizen science datasets collected by anonymous or undirected members of the population is challenging without an equivalent scientifically collected dataset. Comparing similarly structured datasets can identify consistent errors that can be used to correct systematic biases, as was done in this study. The analysis of the active surveillance and citizen science datasets revealed that the free tick testing programme exhibited consistent but addressable participation bias, perhaps manifesting from uneven public awareness of the citizen science programme [30,44] (figure 2). Scientifically collected datasets across large geographical and temporal ranges at sufficiently fine resolutions to appropriately compare with a citizen science dataset are expensive in both time and effort, thus obviating much of the power of citizen science.

The size and expanse of the scientifically collected dataset needed to validate a citizen science dataset could be greatly reduced with *a priori* knowledge about human-relevant factors that may influence the public. Rather than relying on individual-level information to account for data collection biases, as has normally been done with citizen science data (e.g. [45–48]), this study incorporated prior knowledge of the disease system [34] to identify human-relevant, broad-scale factors that dramatically increased the accuracy of the citizen science dataset (figure 3). However, it is highly probable that many factors that may address systematic biases were not investigated (e.g. hospital visits or annual state park visits). Regardless, this study demonstrates the potential value of using population-level, collector-associated factors to account for biases in existing citizen science datasets. Seeking expertise from other fields such as anthropology or sociology will probably identify many additional collector-associated factors that could influence citizen science participation. Including experts who are knowledgeable about both the study system and the human population participating in citizen science-based studies may be essential to realize the power of these datasets.

The human-relevant, broad-scale factors investigated in this study were chosen *a priori* based on both prior studies suggesting their connection to Lyme disease epidemiology and accessibility of the data at appropriate spatial and temporal scales [34]. Although individuals with greater risk for Lyme disease were expected to be more motivated to participate in Lyme disease-related studies (based on behavioural studies from disease immediacy bias [49]), this link was not directly supported by the analyses. That is, local Lyme disease incidence rates paired with the citizen science dataset did not improve estimates of tick abundance (table 1). However, neither nonlinear relationships nor interactions with Lyme disease epidemiology were explored, which may account for additional variation. Nevertheless, the combination of collector-associated factors and public tick submissions as predictors did result in accurate estimates of tick population sizes, although the impact of each individual factor varied considerably. The value of using human-relevant, broad-scale factors to account for systematic errors in citizen science datasets suggests unexplored intersections of social science data and citizen science-based ecological studies.

The Lyme disease system provides a uniquely rich collection of datasets, including multiple estimates of tick abundance through both traditional scientific collections and large-scale citizen science projects. This data-rich environment is ideal for estimating the suitability of large-scale citizen science datasets [50,51]. Further, bias-corrected citizen science datasets can be used to extrapolate tick abundance to nearby states, although these predictions to neighbouring states require validation through active tick collections (figure 4). Similar citizen science datasets are available for the pathogens vectored by these and other ticks across all US states, which can be useful in updating national tick and pathogen maps (e.g. [30]). Although the depth of these datasets is influenced by the public attention associated with Lyme disease, the approach to evaluating citizen science projects can be applied to other systems to capitalize on available citizen science data [52–55]. Identification of factors that correct biases in study systems with ongoing public submission and sample collection could be guided by collector-associated factors indirectly related to the study system.

The ongoing expansion of citizen science data provides unrealized potential to address many of the constraints that pervade large-scale scientific investigations. Effective population surveillance requires frequent, reliable observations over a broad geographical expanse to assess natural fluctuations in population sizes [56]. Public participation in data collection reduces the financial challenges and geospatial limitations to monitor populations more comprehensively. Moreover, delimiting the geographical range of species extends beyond ecological and conservation goals to surveillance for existing and emerging public health threats [57]. Citizen science projects can monitor animal hosts or the microbes they host as early sentinels prior to realizing the consequences of outbreaks of emerging and re-emerging diseases. Worldwide pathogen monitoring has taken on a new significance in light of recent concerns about disease spillovers between wildlife and incidental hosts, including humans.

## Data accessibility

The analytical R code used in this paper is available at Dryad (https://doi.org/10.5061/dryad.v9s4mw6vq). Tick submission data for the citizen science data can be found in Nieto *et al*. [30]. Relevant R code and tick collection data for the NYSDOH data that were previously published can be found at MendeleyData (https://doi.org/10.17632/rtd52gnbyy.1) with further detail in the supplementary material for Tran *et al*. [23].
